# Drug Resistance Spread in 6 Metropolitan Regions, Germany, 2001–2018^1^

**DOI:** 10.3201/eid2610.191506

**Published:** 2020-10

**Authors:** Melanie Stecher, Antoine Chaillon, Christoph Stephan, Elena Knops, Niko Kohmer, Clara Lehmann, Josef Eberle, Johannes Bogner, Christoph D. Spinner, Anna Maria Eis-Hübinger, Jan-Christian Wasmuth, Guido Schäfer, Georg Behrens, Sanjay R. Mehta, Jörg Janne Vehreschild, Martin Hoenigl

**Affiliations:** University of Cologne Faculty of Medicine, Cologne, Germany (M. Stecher, C. Lehmann, J.J. Vehreschild);; University Hospital of Cologne, Cologne (M. Stecher, E. Knops, C. Lehmann, J.J. Vehreschild);; German Center for Infection Research (DZIF), partner site Cologne, Cologne (M. Stecher, C. Lehmann, J.J. Vehreschild);; University of California San Diego, San Diego, CA, USA (A. Chaillon, S.R. Mehta, M. Hoenigl);; University Hospital of Frankfurt, Frankfurt am Main, Germany (C. Stephan, J.J. Vehreschild);; LMU München, Munich, Germany (J. Eberle);; German Center for Infection Research (DZIF), partner site Munich, Munich (J. Eberle, J. Bogner, C.D. Spinner);; Department of the Ludwig-Maximilians-University, Munich (J. Bogner);; Technical University of Munich School of Medicine, Munich (C.D. Spinner);; University of Bonn Medical Center, Bonn, Germany (A.M. Eis-Hübinger);; University Hospital of Bonn, Bonn (J.-C. Wasmuth);; University Hospital Hamburg Eppendorf, Hamburg, Germany (G. Schäfer);; Hannover Medical School, Hannover, Germany (G. Behrens);; German Center for Infection Research (DZIF), partner site Hannover, Hannover, Germany (G. Behrens);; San Diego Veterans Administration Medical Center, San Diego (S.R. Mehta); Medical University of Graz, Graz, Austria (M. Hoenigl)

**Keywords:** HIV transmission, antimicrobial resistance, ART, antiretroviral therapy, mutations, phylogenetic analysis, public health, Germany

## Abstract

We analyzed 1,397 HIV-1 *pol* sequences of antiretroviral therapy–naive patients in a total of 7 university hospitals in Bonn, Cologne, Frankfurt, Hamburg, Hannover, and Munich, Germany. Phylogenetic and network analysis elucidated numerous cases of shared drug resistance mutations among genetically linked patients; K103N was the most frequently shared mutation.

The use of antiretroviral therapy (ART) has shown markedly decreased sickness and death rates in persons living with HIV (PLWH) (*1**–**3*). Meanwhile, the emergence of antimicrobial drug resistance in HIV-1 is raising public health concerns (*4*,*5*). Nationwide estimates of the prevalence of drug resistance mutations (DRMs) are not available in Germany (*6*); the reported prevalence of transmitted HIV-1 DRMs differ across regions and risk groups from 10.4%–17.2%, as described in 2 cohort studies from the German ClinSurv-HIV cohort and the Cologne-Bonn cohort (*6*,*7*).

Information about the dynamics and patterns of HIV transmission within defined areas and communities remains incomplete. Thus, we combined phylogenetic analysis with clinical and sociodemographic data, to determine the spread and dynamics of HIV-1 DRMs in 6 metropolitan regions in Germany, including the cities with the highest rates of new HIV-1 infections in 2017: Munich (17.3/100,000 population), Cologne (13.3/100,000 population), and Frankfurt (12.3/100,000 population), (*8*). We conducted this retrospective study in a cooperative effort of partner sites of the Translational Platform HIV (TP-HIV) (Cologne, Germany) and the University of California, San Diego (San Diego, CA, USA). The study was approved by the local ethics committees of the university hospitals of Bonn, Cologne, Munich, Hannover, Frankfurt, and Hamburg. All study participants gave written informed consent.

## The Study

We analyzed HIV-1 partial *pol* sequences (HXB2 position 2550–3356), obtained as part of clinical routine care, and sociodemographic data of PLWH who received HIV care at the university hospitals of Bonn, Cologne, Frankfurt, Hamburg, and Hannover and at 2 hospitals in Munich during 2001–2018. Patients could participate in the study if they had recently received their diagnosis of HIV-1 and were ART naive; this conservative approach excluded participants for whom the exact start date of ART or history of prior ART was not accurately documented.

Blood samples were collected before ART initiation. We sequenced partial HIV-1 *pol* region as previously described (*7*,*9*). We set the mixed mutation calling threshold at >10%, consistent with Sanger sequencing sensitivity (*10*). We identified major DRMs by using the Stanford University Genotypic Resistance Interpretation HVdb version 8.9, (https://hivdb.stanford.edu). We inferred the genetic transmission network as previously described (*7*,*9*,*11*); we inferred putative linkage for genetic distances <1.5% (*12*) (Appendix).

We performed statistical analyses by using Stata version 14 (StataCorp LP, https://www.stata.com). We applied Fisher exact or χ^2^ test and univariable and multivariable logistic regression models, as appropriate, to determine characteristics that are associated with shared DRM and clustering. A shared DRM was defined as any DRM present in >2 genetically linked persons.

Overall, 1,397 HIV-1 infected participants were included. Most were male (82.9%; 1,158/1,397), originated from Germany (69.6%; 972/1,397), and infected with HIV-1 subtype B (72.8%; 1,017/1,397). The most commonly reported transmission risk was men who have sex with men (MSM) (56.7%; 792/1,397) (Table 1).

**Table 1:** Characteristics of study participants with HIV harboring drug resistance mutations, Germany, 2001–2018*

Characteristic	No. (%) participants	No. (%) with DRMs	No. (%) with shared DRMs†	p value‡
Total	1,397 (100)	248 (17.8)	19 (8.1)	
Age, y				**0.032**
>45	430 (30.8)	82 (19.1)	2 (0.5)	
25–45	856 (61.3)	145 (16.9)	13 (1.5)	
<25	111 (7.9)	21 (18.9)	4 (3.6)	
Sex				0.059
F	239 (17.1)	39 (16.3)	0	
M	1,158 (82.9)	209 (18.0)	19 (1.6)	
HIV subtype				**0.003**
Non-B	380 (27.2)	65 (17.1)	0	
B	1,017 (72.8)	183 (17.9)	19 (1.9)	
Transmission risk§				0.164
HTS	302 (21.6)	48 (15.9)	2 (0.7)	
MSM	792 (56.7)	138 (17.4)	15 (1.9)	
Endemic	133 (9.5)	22 (16.5)	0	
PWID	24 (1.7)	4 (16.7)	1 (4.2)	
Other/Unknown	146 (10.5)	36 (24.7)	1 (0.7)	
Country of origin				0.104
Germany	972 (69.6)	181 (18.6)	17 (1.7)	
Other	373 (26.7)	58 (15.5)	1 (0.3)	
Unknown	52 (3.7)	9 (17.3)	1 (1.9)	
City				0.051
Cologne	582 (41.7)	110 (18.9)	14 (2.4)	
Hamburg	48 (3.4)	9 (18.8)	0	
Bonn	152 (10.9)	22 (14.5)	3 (1.9)	
Frankfurt	215 (15.4)	33 (15.4)	1 (0.5)	
Hannover	169 (12.1)	53 (31.4)	1 (5.9)	
Munich	231 (16.5)	21 (9.1)	0	
Year of HIV-1 diagnosis				0.206
2001–2006	103 (7.4)	14 (13.6)	0	
2007–2012	705 (50.5)	130 (18.4)	13 (1.8)	
2013–2018	589 (42.2)	104 (17.7)	6 (1.0)	

*DRM, drug resistance mutation; endemic, recent immigration from a country with a HIV prevalence >1%; HTS, heterosexuals; MSM, men who have sex with men; PWID, persons who injected drugs. †Shared DRM were defined as any DRM present in >2 genetically linked patients (<1.5% GD) ‡Fisher exact and χ^2^ test were performed as appropriate. Bold text indicates significant results. §Polymorphic mutations are not included in the prevalence of DRMs.

We identified an overall prevalence of any DRM at the time of diagnosis, excluding polymorphic mutations, of 17.8% (95% CI 15.7%–19.8%), 248/1,397 participants. The proportion varied significantly (p<0.001) by region, ranging from 9.1% (95% CI 5%–13%; 21/231) in Munich, up to 31.4% (95% CI 24%–38%; 53/169) in Hannover. Resistance mutations associated with nucleoside reverse transcriptase inhibitors (NRTIs) (172/1,397; 12.3%) were most frequent, followed by nonnucleoside reverse transcriptase inhibitors (NNRTIs) (124/1,397; 8.9%). The most common single mutations related to NNRTIs were K103N (31/124; 25.0%), and G190A (8/124; 6.5%). Of the NRTI resistance mutations, M41L (25/172; 14.5%), and T215S (18/172; 10.5%) were most frequently observed (Table 2).

**Table 2:** Proportion of identified drug resistance mutations in newly infected antiretroviral-naive patients with HIV-1, Germany, 2001–2018*

Mutation	Bonn, no. (%)	Cologne, no. (%)	Frankfurt, no. (%)	Hamburg, no. (%)	Hannover, no. (%)	Munich, no. (%)
NRTI						
T215FY	3 (1.21)	19 (7.66)	4 (1.61)	1 (0.40)	19 (7.66)	6 (2.42)
M41L	1 (0.40)	13 (5.24)	3 (1.21)	1 (0.40)	6 (2.42)	1 (0.40)
D67GNS	3 (1.21)	13 (5.24)	0	0	3 (1.21)	1 (0.40)
K219ERQ	3 (1.21)	7 (2.82)	1 (0.40)	0	3 (1.21)	2 (0.81)
M184IV	0	9 (3.63)	0	0	3 (1.21)	1 (0.40)
A62V	0	1 (0.40)	1 (0.40)	1 (0.40)	3 (1.21)	1 (0.40)
E44D	0	4 (1.61)	1 (0.40)	0	2 (0.81)	0
K70RT	0	4 (1.61)	0	0	2 (0.81)	0
L210W	1 (0.40)	2 (0.81)	0	0	3 (1.21)	0
T69D	1 (0.40)	4 (1.61)	0	0	1 (0.40)	0
F77L	0	0	0	0	4 (1.61)	0
L74V	0	4 (1.61)	0	0	0	0
K65R	0	2 (0.81)	0	0	1 (0.40)	0
V75AIM	0	3 (1.21)	0	0	0	0
NNRTI						
E138A†	7 (2.82)	21 (8.47)	11 (4.44)	1 (0.40)	6 (2.42)	3 (1.21)
K103ENT	7 (2.82)	16 (5.13)	5 (2.02)	2 (0.81)	4 (1.61)	3 (1.21)
V179DEF	0	11 (4.44)	4 (1.61)	2 (0.81)	8 (3.23)	5 (2.02)
G190AERS	2 (0.81)	9 (3.63)	0	0	2 (0.81)	1 (0.40)
Y188LHC	2 (0.81)	4 (1.61)	0	0	2 (0.81)	1 (0.40)
L100IV	0	2 (0.81)	3 (1.21)	0	3 (1.21)	0
Y181C	1 (0.40)	3 (1.21)	0	0	1 (0.40)	1 (0.40)
V108I	1 (0.40)	3 (1.21)	0	0	1 (0.40)	0
P225H	1 (0.40)	3 (1.21)	0	0	0	0
V106AIM	0	4 (1.61)	0	0	0	0
M230MI	0	2 (0.81)	0	0	0	0
A98AG	0	1 (0.40)	0	0	0	0
F227FL	0	1 (0.40)	0	0	0	0
H221HY	0	1 (0.40)	0	0	0	0
K101E	0	0	0	0	1 (0.40)	0
K238T	0	1 (0.40)	0	0	0	0
PI						
L90M	0	0	0	0	5 (2.02)	1 (0.40)
M46I	0	0	1 (0.40)	1 (0.40)	2 (0.81)	0
I84V	0	0	0	0	2 (0.81)	0
I47V	0	0	0	1 (0.40)	0	0
L90LM	0	0	0	0	1 (0.40)	0
M46L	0	0	1 (0.40)	0	0	0
V82L	0	0	0	0	0	1 (0.40)

*Data are presented by each city’s university hospital as absolute numbers and percentages. No resistances to integrase strand transfer inhibitors were identified. NNRTI, nonnucleoside reverse transcriptase inhibitors; NRTI, nucleoside reverse transcriptase inhibitors; PI, protease inhibitors. †E138A was not included in the drug resistance mutation/transmitted drug resistance mutation rate of our study population.

**Figure 1:**
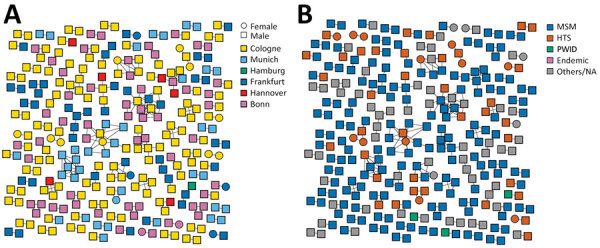
Transmission network analysis by sex and location (A) and by characteristic (B) for 1,397 patients with HIV, Germany, 2001–2018. Endemic, recent immigration from a country with HIV prevalence >1%; HTS, heterosexual patient; MSM, men who have sex with men; NA, not available; PWID, persons who inject drugs.

Transmission network analyses revealed that 20.7% (289/1,397) of participants had a putative linkage forming 102 transmission clusters. The largest cluster included 12 participants, mostly MSM from Bonn, Cologne, Munich, and Frankfurt (Figure 1, panels A, B). Participants <25 years and 25–45 years of age were significantly more likely to cluster compared with participants >45 years (<25 years, adjusted OR [aOR] 4.38, 95% CI 2.55–7.52, p<0.001; 25–45 years, aOR 1.91, 95% CI 1.36–2.678; p<0.001). Participants infected with HIV-1 subtype B were more likely to cluster than those with non-B subtype (aOR 4.05, 95% CI 2.37–6.90; p<0.001). Geospatial distribution differed; participants from Bonn were linked significantly more often than those from Cologne (aOR 1.63; 95% CI 1.06–2.49; p = 0.025), even though the cities are geographically close (Appendix Table).

**Figure 2:**
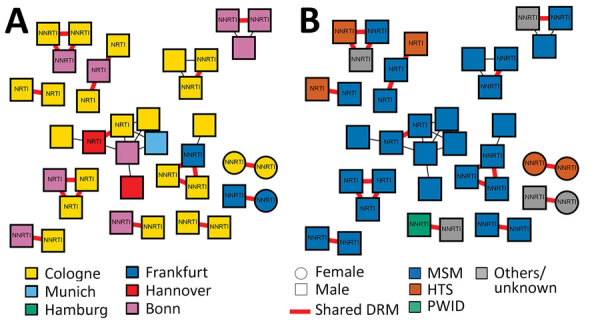
Presence of drug resistance mutations by location (A) and by risk factor (B) for 1,397 patients with HIV, Germany, 2001–2018. DRM, drug resistance mutation; HTS, heterosexual; MSM, men who have sex with men; NNRTI, nonnucleoside reverse transcriptase inhibitor; NRTI, nucleoside reverse transcriptase inhibitor; PWID, persons who inject drugs.

The prevalence of transmitted DRM was comparable in clustering (47/289, 16.3%) and nonclustering (201/1,108; 18.1%) participants (p = 0.46) (Appendix Table). Of the 47 sequences harboring DRM, 19 (40.4%) were preferentially shared by participants living predominantly in Cologne (14/19, 73.7%) and Bonn (3/19, 15.8%) (Figure 2, panel A) and by participants reporting MSM as main risk factor (15/19; 78.9%) (Figure 2, panel B). Younger age (<25 years) was associated with a higher proportion of shared DRM (3/11; 3.5%) compared with older age (24–45 years, 13/856 [1.5%]; >45 years, 2/430 [0.5%]) (Table 1).

The most frequently observed putatively shared DRM was K103N, detected in 9/19 (47.4%) participants forming 4 distinct clusters, predominantly originating from Cologne (7/9, 77.8%). The second most common shared DRM was D67N, found in 6/19 (31.6%) participants from Cologne and Bonn.

## Conclusions

The increasing prevalence of DRMs in PLWH has become a serious matter of concern for clinicians and public health entities (*4*). In our study, we observed a 17.8% prevalence of DRMs, higher than in previous studies (*6*,*7*). The proportion of NNRTI resistance mutations was 8.9%, which is potentially associated with the common use of NNRTI in first-line ART regimens. K103N represented one quarter of NNRTI resistance mutations, reducing susceptibility to the first-generation drugs nevirapine and efavirenz (*13*). Transmission network analyses revealed that K103N was the most frequently shared DRM.

K65R, K70RT, and M184IV were the most common of the NRTI resistance mutations we observed, particularly among the risk group of MSM living in Cologne and Hannover, indicating potential resistance to preexposure prophylaxis (PrEP) with tenofovir/emtricitabine. Such resistance might be an upcoming challenge as PrEP use increases. Monitoring for HIV infections with these mutations is of utmost importance for preventing an epidemic among high-risk PrEP users; one mitigation is to consider alternative PrEP regimens in regions with high resistance.

Our study had several limitations. First, our sample population could have been biased because participants were not randomly selected; our dataset was limited to ART-naive patients who received an HIV diagnosis at 7 university hospitals during 2001–2018. Although we know no reason why a university hospital setting would not be representative of the region, it is possible that populations treated outside these centers may have different transmission networks and risks; results are not generalizable to the entire regions or nationwide. Second, mixing of heterosexual patients and MSM in clusters may be due to missing single or multiple risk factors. Thus, their role could not be represented in the transmission networks. Third, we have not tested clinical correlates and drug resistance; the clinical relevance was inferred from the Stanford database.

In summary, we found that the overall rate of DRM was high in Germany. Network analysis elucidated cases of shared DRMs among genetically linked persons, mainly in MSM-dominated clusters. Our findings highlight regional differences and illustrate the need to test MSM, especially younger men, for HIV regularly and to evaluate local HIV programs and adapt screening and treatment strategies to local epidemics.

Appendix Additional information about drug resistance in patients with HIV-1 in 6 metropolitan cities, Germany.
