# Supplementary material for: Drug Resistance Spread in 6 Metropolitan Regions, Germany, 2001–2018
Source: Emerg Infect Dis. 2020 Oct;26(10):2439–43. doi: 10.3201/eid2610.191506 (PMC7510719; doi:10.3201/eid2610.191506)
Supplement: Appendix — Additional information about drug resistance in patients with HIV-1 in 6 metropolitan cities, Germany. [file 19-1506-Techapp-s1.pdf]

Article DOI: https://doi.org/10.3201/eid2610.191506

Pretreatment HIV Drug Resistance Spread in 6 Metropolitan Regions, Germany, 2001–2018

Appendix

Sequence and Transmission Network Analysis

Analysis was performed using one HIV-1 partial *pol* sequence from each unique individual (HXB2 position 2550–3356) obtained at the first HIV clinic visit. The Subtype Classification using Evolutionary Algorithms (SCUEAL) program was used to subtype all sequences and the HIV-TRACE software (HIV TRAnsmission Cluster Engine; www.hivtrace.org) was used to infer putative transmission clusters (*1*,*2*). All partial HIV-1 *pol* sequences were aligned to the HXB2 reference sequence. Putative transmission links (i.e., edges) were inferred when 2 sequences (i.e. nodes) had a Tamura-Nei 93 genetic distance of $\leq$1.5% (*3*). The $\leq$1.5% threshold has been established as a standard in the field, and is based on study findings showing that within mono-infected persons, *pol* sequences typically do not diverge >1% during the first 10 years of infection. All nucleotide ambiguities were resolved and only sequences with <1.5% diversity were retained. Multiple linkages were then combined into putative clusters. Clusters comprised of only 2 linked individuals were identified as dyads. We assessed potential confounding effects of convergent evolution for drug resistances by repeating our analysis after we have excluded 48 codon positions in protease and reverse transcriptase, which are associated with drug resistance (*4*,*5*).

Appendix Table. Demographics and characteristics of patients with HIV and results of univariate and multivariate transmission analysis, Germany, 2001–2018*

Characteristic	Nonclustering no. (%)	Clustering no. (%)	Univariate analysis OR 95% CI	Univariate analysis p-value	Multivariable analysis OR 95% CI	Multivariable analysis p-value†
Total	1,108 (79.3)	289 (20.7)				
Age, y						
>45	367 (85.3)	63 (14.7)	Ref		Ref	
25–45	671 (78.4)	185 (21.6)	1.60 (1.18–2.20)	**0.003**	1.91 (1.36–2.68)	**<0.001**
<25	70 (63.1)	41 (36.9)	3.41 (2.14–5.45)	**<0.001**	4.38 (2.55–7.52)	**<0.001**
DRM						
No	907 (78.2)	242 (21.1)	Ref			
Yes	201 (84.8)	47 (19.0)	0.88 (0.62–1.24)	0.457		
Sex						
F	222 (92.90)	17 (7.1)	Ref		Ref	
M	886 (76.5)	272 (23.5)	4.01 (2.40–6.69)	**<0.001**	1. 62 (0.85–3.06)	0.140
HIV subtype						
non-B	360 (94.7)	20 (5.3)	Ref		Ref	
B	748 (73.5)	269 (26.5)	6.47 (4.04–10.37)	**<0.001**	4.05 (2.37–6.90)	**<0.001**
Transmission risk						
HTS	253 (83.8))	49 (16.2)	Ref		Ref	
MSM	580 (73.2)	212 (26.8)	1.89 (1.34–2.66)	**<0.001**	0.89 (0.58–1.38)	0.623
Endemic	133 (100)	-	NA		NA	
PWID	21 (87.5)	3 (12.5)	0.74 (0.21–2.57)	0.633	0.59 (0.15–2.29)	0.586
Others/Unknown	121 (82.9)	25 (17.1)	1.07 (0.63–1.81)	0.810	0.92 (0.50–1.69)	0.922
Country of origin						
Germany	733 (75.4)	239 (24.6)	Ref		Ref	
Other	333 (89.3)	40 (10.7)	0.37 (0.26–0.53)	**<0.001**	0.70 (0.47–1.06)	0.095
Unknown	42 (80.8)	10 (19.2)	0.73 (0.36–1.48)	0.382	0.88 (0.40–1.90)	0.736
City						
Cologne	444 (76.3)	138 (47.8)	Ref		Ref	
Hamburg	47 (97.9)	1 (2.1)	0.07 (0.01–0.50)	0.008	NA	
Bonn	94 (61.8)	58 (38.2)	1.99 (0.62–1.31)	**<0.001**	1.63 (1.06–2.49)	**0.025**
Frankfurt	168 (78.1)	47 (21.9)	0.90 (0.62–1.31)	0.583	0.81 (0.52–1.25)	0.342
Hannover	163 (96.4)	6 (3.6)	0.12 (0.05–2.73)	**<0.001**	0.10 (0.01–0.36)	**<0.001**
Munich	192 (83.1)	39 (16.9)	0.65 (0.44–0.97)	**0.034**	0.62 (0.40–0.94)	**0.025**
Year of HIV-1 diagnosis						
2001–2006	91 (88.3)	12 (11.7)	Ref		Ref	
2007–2012	566 (80.3)	139 (19.7)	1.86 (0.99–3.49)	0.053	2.59 (1.34–5.00)	**0.005**
2013–2018	451 (76.6)	138 (23.4)	2.32 (1.23–4.36)	**0.009**	3.36 (1.71–6.61)	**<0.001**

*The univariable and multivariable logistic regression model determined associating factors on clustering persons (clustering yes/no as dependent variable). Endemic, recent immigration from a country with HIV prevalence >1%; HTS, heterosexuals; MSM, men who have sex with men; NA, not applicable; PWID, persons who inject drugs; Ref, reference category for univariable and multivariable regression model.
†Univariate and multivariable logistic regression model. Bold text indicates significant results. We used the enter method to reach the best model that explained the data. Our final multivariable model was adjusted for age, sex, HIV subtype, reported risk group, country of origin, city of residence, and year of HIV-1 diagnosis.

References

1. Kosakovsky Pond SL, Weaver S, Leigh Brown AJ, Wertheim JO. HIV-TRACE (TRAnsmission Cluster Engine): a tool for large scale molecular epidemiology of HIV-1 and other rapidly evolving pathogens. Mol Biol Evol. 2018;35:1812–9. PubMed https://doi.org/10.1093/molbev/msy016

2. Kosakovsky Pond SL, Posada D, Stawiski E, Chappey C, Poon AF, Hughes G, et al. An evolutionary model-based algorithm for accurate phylogenetic breakpoint mapping and subtype prediction in HIV-1. PLOS Comput Biol. 2009;5:e1000581. PubMed https://doi.org/10.1371/journal.pcbi.1000581

3. Tamura K, Nei M. Estimation of the number of nucleotide substitutions in the control region of mitochondrial DNA in humans and chimpanzees. Mol Biol Evol. 1993;10:512–26. PubMed

4. Wheeler WH, Ziebell RA, Zabina H, Pieniazek D, Prejean J, Bodnar UR, et al.; Variant, Atypical, and Resistant HIV Surveillance Group. Prevalence of transmitted drug resistance associated mutations and HIV-1 subtypes in new HIV-1 diagnoses, U.S.—2006. AIDS. 2010;24:1203–12. PubMed https://doi.org/10.1097/QAD.0b013e3283388742

5. Wertheim JO, Kosakovsky Pond SL. Purifying selection can obscure the ancient age of viral lineages. Mol Biol Evol. 2011; 28:3355–3365. https://doi.org/10.1093/molbev/msr170
